# Residual Compressive Strength Prediction Model for Concrete Subject to High Temperatures Using Ultrasonic Pulse Velocity

**DOI:** 10.3390/ma16020515

**Published:** 2023-01-05

**Authors:** Wonchang Kim, Hyeonggil Choi, Taegyu Lee

**Affiliations:** 1 Department of Fire and Disaster Prevention, Semyung University, Jecheon-si 27136, Republic of Korea; 2 School of Architecture and Civil Engineering, Kyungpook National University, Daegu 41566, Republic of Korea

**Keywords:** high temperature, ultrasonic pulse velocity, compressive strength, type of coarse aggregate, water-binder, prediction model

## Abstract

This study measured and analyzed the mechanical properties of normal aggregate concrete (NC) and lightweight aggregate concrete (LC) subjected to high temperatures. The target temperature was set to 100, 200, 300, 500, and 700 °C, and W/C was set to 0.41, 0.33 and 0.28 to evaluate high temperature properties at various intensities. Measurement parameters included mass loss, compressive strength, ultrasonic pulse velocity (UPV), and elastic modulus. We compared the residual mechanical properties between the target and preheating temperatures (20 °C) and then analyzed the correlation between UPV and compressive strength. According to the research findings, after exposure to high temperatures, LC demonstrated a higher mass reduction rate than NC at all levels and exhibited higher residual mechanical properties. The results of analyzing the correlation between compressive strength and UPV for concrete subjected high temperatures were very different from the compressive strength prediction equation previous proposed at room temperature, and the error range of the residual strength prediction equation considering W/C was reduced.

## 1. Introduction

After exposure to fire, the main structural members of a concrete building subjected to high-temperature exhibit durability degradation. Thus, evaluating the safety of such degraded concrete is critical because strength degradation may result in the collapse of large buildings with relatively large members. In addition, decisions based on accurate safety evaluation, such as proper repair and demolition, have positive economic effects [1,2,3,4,5]. In particular, high-rise buildings may have problems owing to long-term and continuous loads [6,7,8,9]. Research on lightweight concrete combined with lightweight aggregate has been actively conducted to address these issues [10,11].

Researchers have used non-destructive testing (NDT) methods to predict strength. Among NDT methods, the ultrasonic pulse velocity(UPV) method was used in bridge engineering and earthquake resistance engineering of buildings, and some researchers conducted a fire damage evaluation study using UPV method [12,13]. Roufael et al. [14] investigated normal aggregate concrete (NC) and LC mixed with limestone, expanded shale, and expanded clay as aggregates at high temperatures. They reported that while LC had higher residual compressive strength and UPV than NC, the latter had higher residual density. They also reported improved results in LC compared to NC using scanning electron microscope (SEM) analysis of the interface between paste and aggregate. Yao et al. [15] investigated concrete mixed with gravel and shale ceramsite aggregates at high temperatures and classified shale ceramsite aggregates according to the substitution rate of the gravel aggregate. NC and LC showed the same behavior for compressive strength and UPV as the temperature increased; however, the residual compressive strength and residual UPV of LC were higher than those of NC. Additionally, Yao et al. [15] proposed a compressive strength prediction equation using UPV, which had a correlation coefficient (R^2^) of approximately 0.96.

However, in the case of the previously proposed compressive strength prediction model for concrete subjected to high temperature using UPV, it is somewhat insufficient to consider various strength ranges. Concrete exhibits different strength reduction properties in various strength ranges, i.e., various W/C, and experiments in diverse variables are required to accurately and quantitatively evaluate these properties using UPV. In addition, since different high-temperature properties appear due to the influence of the mixed coarse aggregate, it is estimated that a study of concrete mixed with various aggregates is necessary to quantitatively evaluate this.

In this study, the mechanical properties of NC and LC after exposure to high temperatures (100, 200, 300, 500, and 700 °C) were evaluated at various W/C ratios. Furthermore, after exposure to high temperatures, strength prediction equations were proposed after analyzing the correlation between the compressive strength of concrete and UPV.

## 2. Materials and Methods

### 2.1. Materials

Table 1 shows the physical properties of the materials used in this study (cement, coarse aggregate, fine aggregate, and admixture). Table 2 lists the chemical composition of Type I ordinary Portland cement used in this study.

### 2.2. Experimental Program and Concrete Mix Proportion

**Figure 1:**
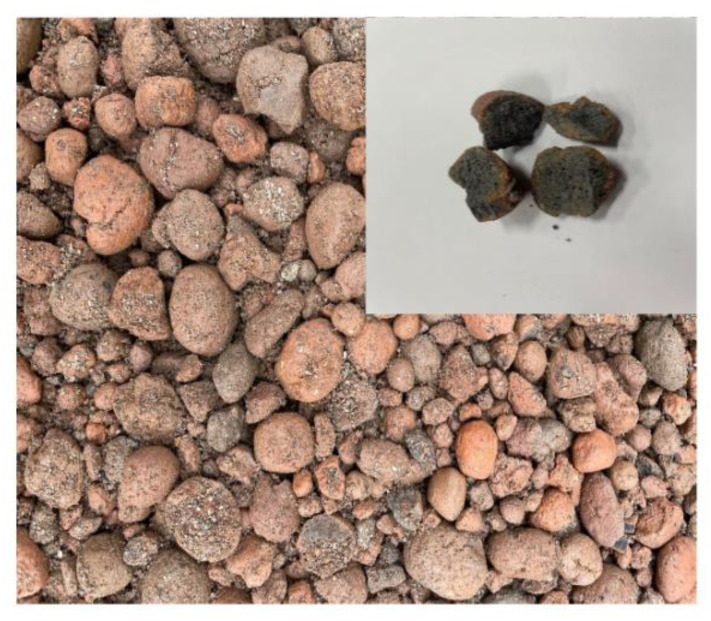
Sample of coal ash lightweight aggregate.

Table 3 shows the experimental program. Cylindrical specimens with a diameter of 100 mm and a height of 200 mm were prepared. Regarding coarse aggregate, crushed granite aggregate was used for NC and coal ash lightweight aggregate for LC (Figure 1) [18]. In the case of lightweight aggregates, after 24 h ‘pre-wetting’, it was dried at room temperature of about 12 h and then mixed.

Target strengths of 30, 45, and 60 MPa were set for NC and LC specimens, respectively. After demolding, the specimens underwent water curing for 28 days before being cured for 91 days at a room temperature of 20 ± 2 °C and humidity of 60 ± 5%.

**Figure 2:**
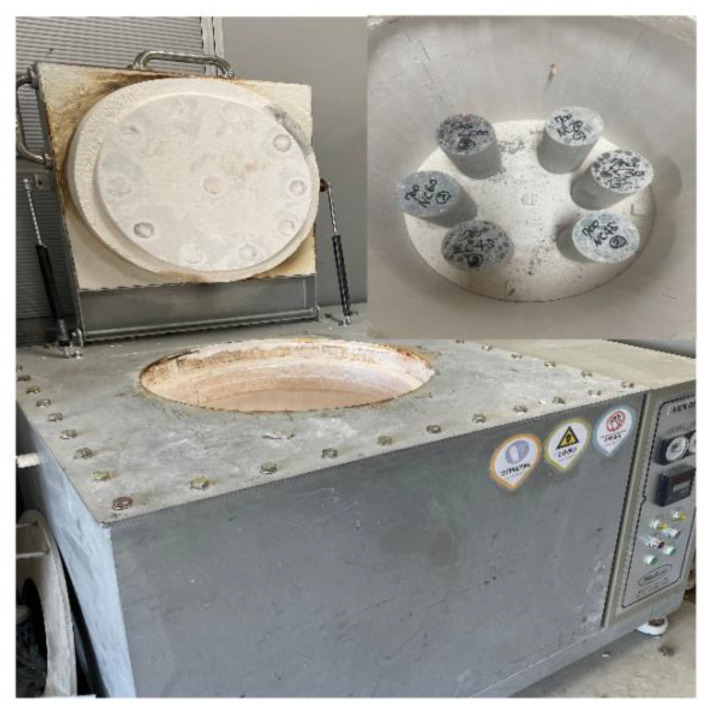
Electric furnace.

**Figure 3:**
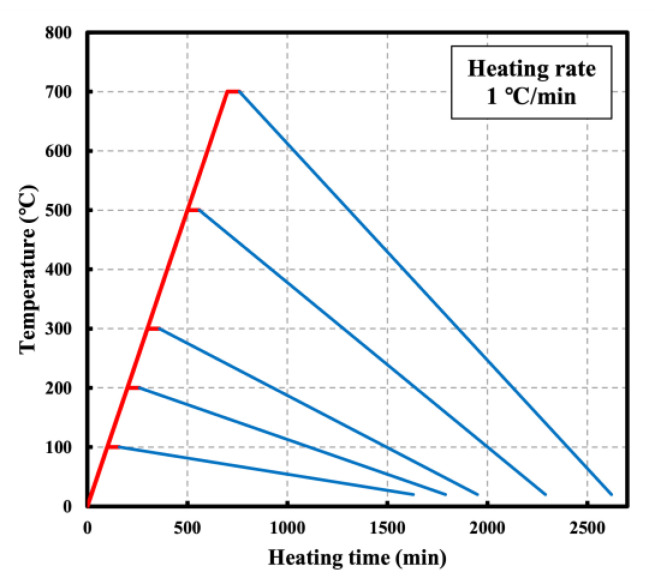
Heating rate.

Figure 2 shows the electric furnace used in the experiment. The target temperatures were set to 23, 100, 200, 300, 500, and 700 °C, with a heating rate of 1 °C/min based on RILEM 129-MHT (Figure 3). The temperature was maintained at the target level for 60 min to match the internal and external temperatures of the specimen.

After heating, mechanical properties were measured after cooling at room temperature for 24 h. The mass loss (%), compressive strength (MPa), UPV (km/s), and elastic modulus (GPa) were set as measurement items, and residual mechanical properties were compared with those before heating (20 °C). The results of the mechanical properties averaged three specimens. The W/C ratio was used to analyze the correlation between the compressive strength of NC and LC after exposure to high temperature and UPV.

Table 4 shows the mix proportions of NC and LC. The W/C ratio was set to 0.41, 0.33 and 0.28 for developing the set target strengths. To analyze the effects of the coarse aggregates of NC and LC on UPV, the cement and water contents per unit volume of concrete and the sand-aggregate (S/a) ratio(volume-based) were all set to the same values except for the coarse aggregates.

### 2.3. Test Methods

**Figure 4:**
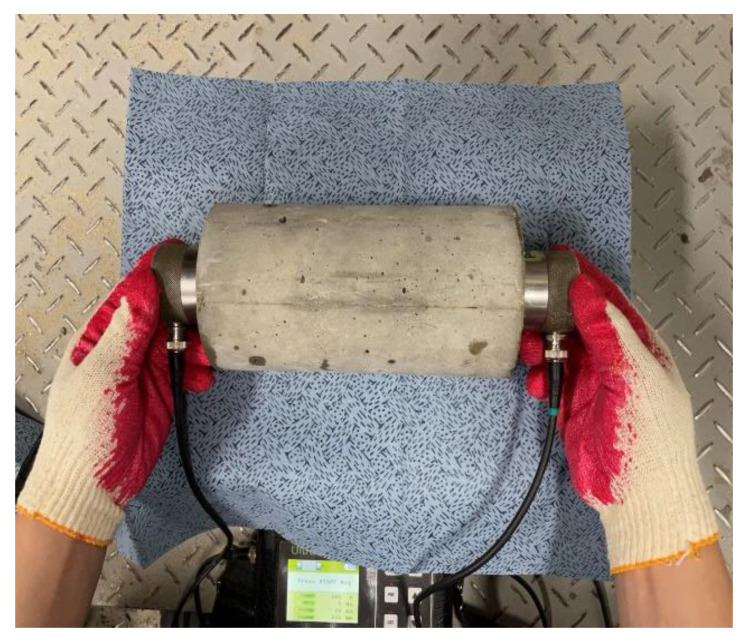
Ultrasonic pulse velocity test [22].

Table 5 shows the testing method for mechanical properties. The compressive strength and the elastic modulus tests were conducted in accordance with ASTM C39/C39M [19] and ASTM C469 [20], respectively. Figure 4 shows the UPV measurement environment, performed in accordance with ASTM C597 [21]. UPV was calculated using Equation (1), as shown in Table 5.

## 3. Results and Discussion

### 3.1. Mechanical Properties of NC and LC after High Temperature

#### 3.1.1. Mass Loss on NC and LC after High Temperature

**Figure 5:**
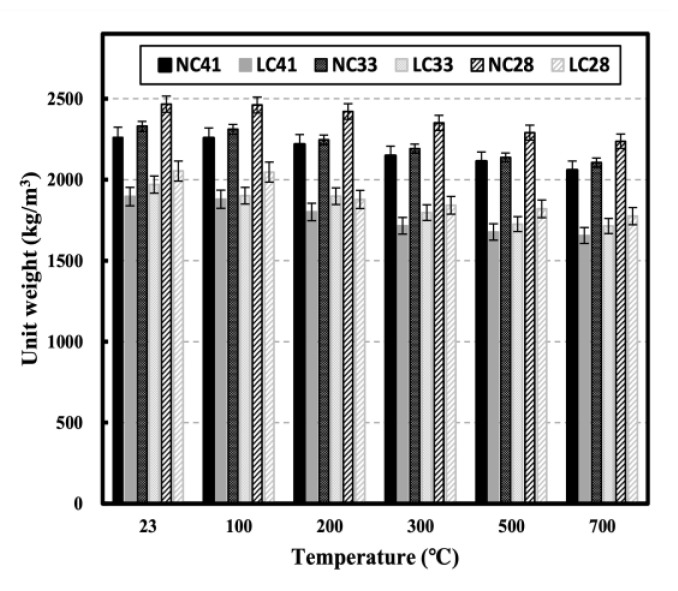
Unit weight of NC and LC after high temperature.

Figure 5 shows the unit weights of NC and LC after exposure to high temperatures. At room temperature (20 °C), the unit weight of NC and LC increased as the W/C ratio decreased, owing to an increase in cement content. Additionally, LC exhibited a lower unit weight than NC at the same W/C ratio, possibly because the coal ash aggregate used in LC has a lower density than the crushed granite aggregate used in NC (Table 1). The unit weight decreases as the temperature increases owing to the discharge of water vapor and free water inside the specimen [23,24]. The influence of the broken fragments of the specimen caused by serious damage to the matrix inside the concrete at 700 °C can also be considered.

**Figure 6:**
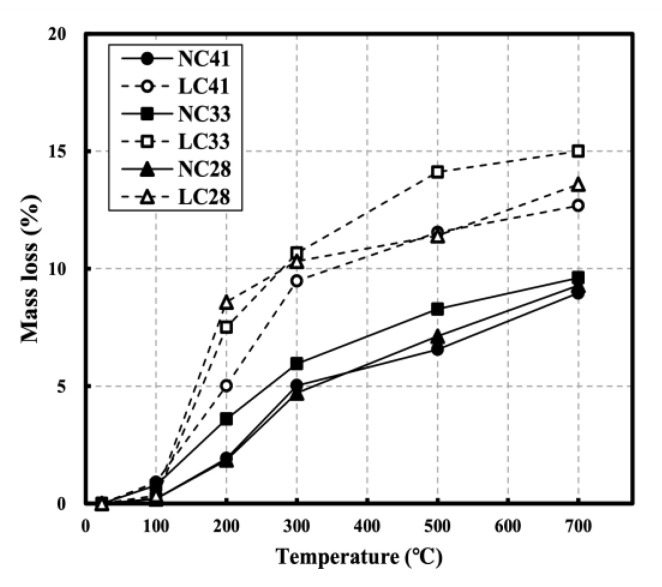
Mass loss of NC and LC after high temperature.

Figure 6 shows the mass loss of NC and LC after exposure to high temperatures. The degree of mass loss at the target temperature based on the mass at room temperature (20 °C) can be observed. At 100 °C, a low mass loss of 1% or less was observed from all specimens. At 200 °C, NC showed a mass loss of less than 5%, while LC showed a mass loss of at least 5%. At 300 °C, NC showed a mass loss of approximately 5%, whereas LC showed a mass loss of approximately 10%. The temperature range of 100–300 °C showed the highest mass loss increase rate. This appears to be due to the dehydration of gypsum in the 100–200 °C range, the large dehydration reaction of ettringite from before 100 °C to approximately 200 °C, and the dehydration of C-S-H, which is the main component of the cement matrix, in the 100–300 °C range. No significant mass loss increase rates were observed for temperatures above 300 °C. At 700 °C, the mass loss was approximately 9.29% for NC and 13.76% for LC, possibly because the lightweight aggregate has a higher absorption rate than the normal aggregate because it is more porous. According to Saridemir et al., the high mass loss up to 400 °C is caused by the evaporation of physically bonded water in the matrix and the mass loss above 400 °C is slow because it is mainly affected by the decomposition of calcium hydroxide [4,25,26]. Therefore, it is concluded that the evaporation of physically bonded water significantly impacts mass loss.

#### 3.1.2. Compressive Strength on NC and LC after High Temperature

**Figure 7:**
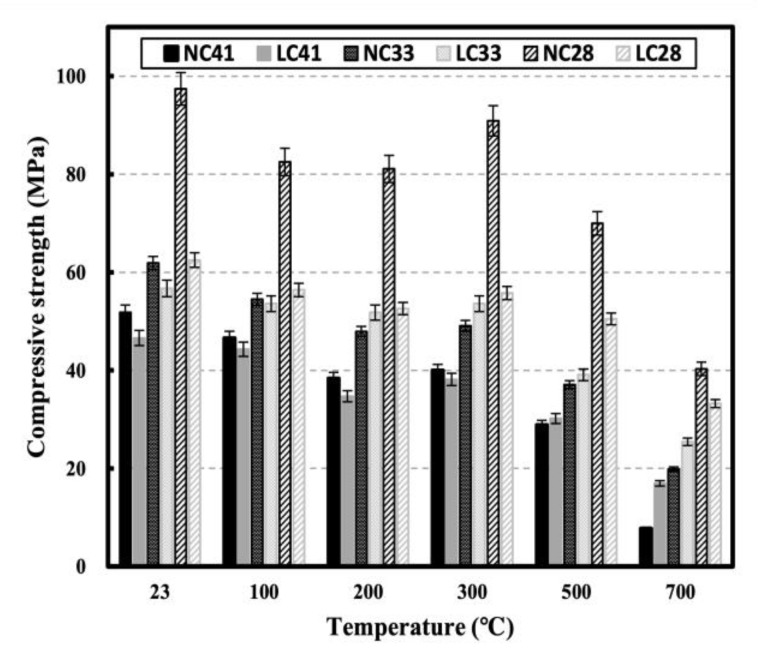
Compressive strength of NC and LC after high temperature.

**Figure 8:**
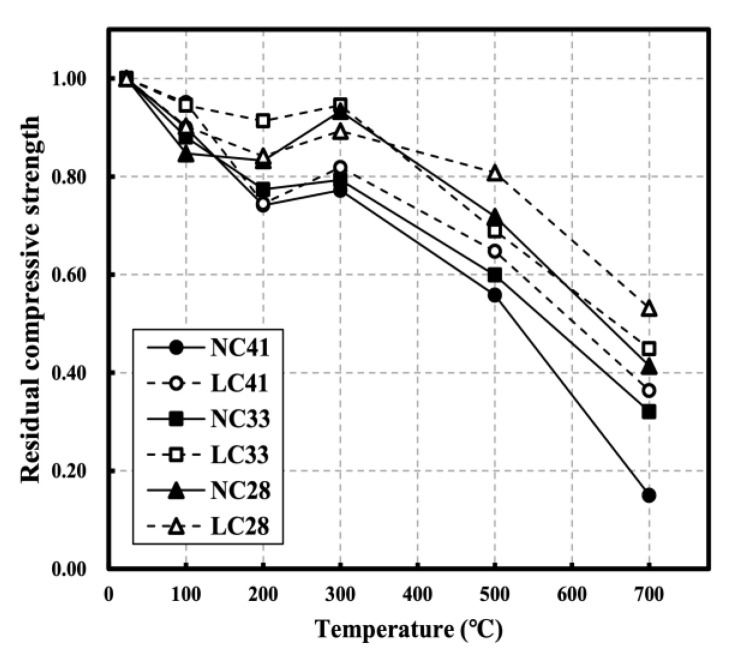
Residual compressive strength of NC and LC after high temperature [22].

Figure 7 and Figure 8 show the compressive strength of NC and LC and the residual compressive strength following exposure to high temperatures, respectively. At 100 °C, the strength decreased by approximately 16% for NC and 10% for LC. Strength degradation was observed up to 200 °C, followed by a slight strength recovery between 200 and 300 °C. According to Lee et al., the vapor pressure and thermal expansion of aggregates generally affect the strength recovery at 200 to 300 °C [27,28,29,30]. Roufael et al. reported slight strength loss up to 150 °C and slight strength increase in the 150–300 °C range [14,31,32,33]. Saridemir et al. reported that the escape of water from the pores of the cement gel increases the matrix strength of cement [25,34,35,36].

Strength continuously decreased at temperatures above 300 °C, and when they exceeded 500 °C, LC41 had a higher compressive strength than NC41, and both NC41 and LC41 had residual compressive strengths of 0.56 and 0.65, respectively. At 700 °C, LC41 (0.36) had a higher residual compressive strength than NC41 (0.15). The compressive strength of LC33 was consistently higher than that of NC33 at temperatures above 200 °C.

The residual compressive strengths of NC33 and LC33 were 0.60 and 0.69 at 500 °C and 0.32 and 0.45 at 700 °C, respectively. NC28 demonstrated greater compressive strength than LC28 at all temperatures, but after 500 °C, the residual compressive strength of LC28 tended to be higher than that of NC28. The residual compressive strengths of NC28 and LC28 were 0.72 and 0.81 at 500 °C and 0.45 and 0.53 at 700 °C, respectively. At all levels, the residual compressive strength increased as the W/C ratio decreased, and at temperatures above 300 °C, the residual compressive strength of LC tended to be higher than that of NC. According to Cakir et al., porous lightweight aggregate tends to have a low thermal expansion owing to internal pores, which improve interfacial transition zone (ITZ) cracks between the aggregate and mortar and is the main cause of concrete strength degradation [10,37,38]. According to Lim et al., coal-ash aggregate forms many internal pores because it is favorable for the formation of a film generated by the melting of materials that constitute the aggregate, as K_2_O and Na_2_O components involved in the formation of a glassy film on the surface of the aggregate are distributed evenly, indicating that coal-ash components are favorable for porosity formation [39]. Roufael et al. used ITZ photographs of degraded aggregate and paste obtained using SEM to demonstrate the excellent thermal resistance of LC compared with NC. They also reported that the stress in the ITZ between the paste and aggregate was increased because the thermal expansion of lightweight aggregate was smaller than that of normal aggregate; thus, the strain between the mortar and aggregate was limited [14,40,41].

#### 3.1.3. Ultrasonic Pulse Velocity on NC and LC after High Temperature

**Figure 9:**
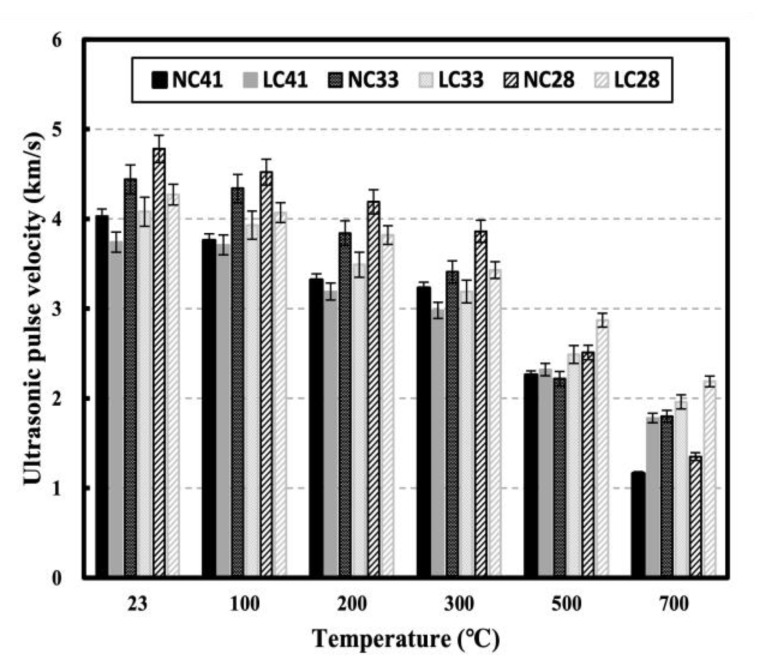
Ultrasonic pulse velocity of NC and LC. after high temperature.

Figure 9 shows the UPV of NC and LC in relation to temperature. At 20 °C, UPV was higher than 4.0 km/s except for LC41, and NC exhibited higher UPV than LC at the same W/C ratio. NC showed higher UPV than LC up to 300 °C, but after 500 °C, LC exhibited higher UPV than NC. NC28 had the highest UPV (4.75 km/s) at 20 °C, but its UPV at 700 °C (1.35 km/s) was similar to that of NC41. In NC28, which has a higher cement content than other specimens, the decomposition rate of C-S-H, which are the main components of the cement matrix, peaks at 700 °C and more cracks and pores occur, significantly affecting the reduction in UPV, which is sensitive to cracks and pores. Additionally, according to Xue et al., coarse aggregate in high-strength concrete reduces the heat absorbed in the hydrate decomposition reaction, and NC28 is demonstrated to have a greater impact on matrix damage than NC41 and NC33 because the content of coarse aggregate is smaller [42].

**Figure 10:**
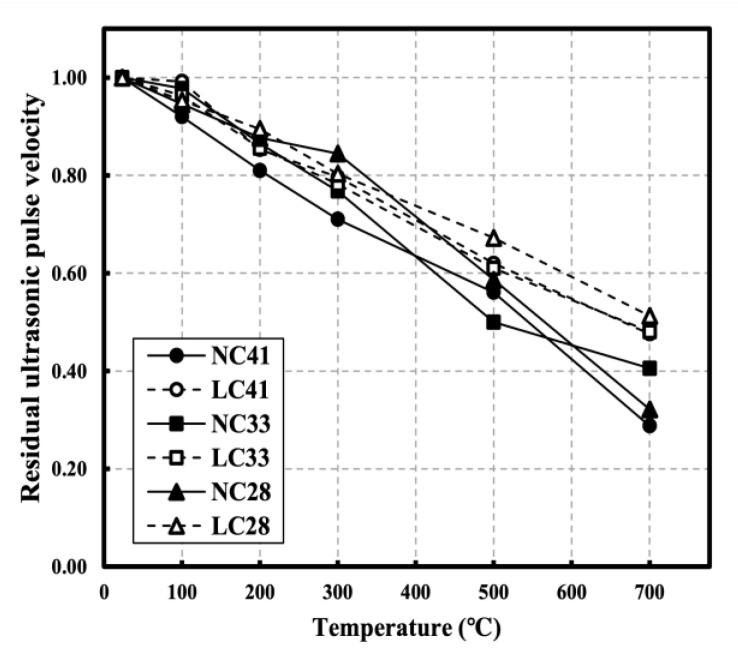
Residual ultrasonic pulse velocity of NC and LC after high temperature [22].

Figure 10 shows the residual UPV in relation to temperature. Residual UPV tended to linearly decrease compared to compressive strength, suggesting that the influence of the microcracks and pores caused by the collapse of the matrix with increasing temperature is more significant than the influence of strength on UPV. All specimens had similar residual UPV at 100 °C, whereas at temperatures below 300 °C, NC41 showed the lowest residual UPV, which was not significantly different compared to other levels. At temperatures above 300 °C, the residual UPV of LC tended to be higher than that of NC at the same W/C ratio. At 500 °C, the residual UPVs of NC41, NC33, and NC28 were 0.56, 0.50, and 0.59, respectively, while those of LC41, LC33, and LC28 were 0.62, 0.61, and 0.67, respectively. At 700 °C, the residual UPVs of NC41, NC33, and NC28 were 0.29, 0.41, and 0.32, respectively, while those of LC41, LC33, and LC28 were 0.48, 0.48, and 0.51, respectively. At 500 °C, the residual UPV of LC was approximately 0.1 higher than that of NC at all W/C ratios. At 700 °C, the residual UPV of LC was significantly different (approximately 0.19 higher) from that of NC at W/C ratios of 0.41 and 0.28. As the temperature increases, the materials inside the specimens exhibit different thermal properties. Generally, cement paste slightly expands at 100 °C before shrinking at higher temperatures, whereas aggregate expands as the temperature increases. These processes result in microcracks and a weakening of ITZ between the paste and aggregate. However, coal-ash lightweight aggregate with porosity improves ITZ between the paste and aggregate because it has lower thermal expansion than normal aggregate and fewer microcracks than concrete mixed with normal aggregate [14,37,38].

The arrival time of LC is improved owing to relatively small cracks [43]. According to Roufael et al., the residual UPV of LC is higher than that of NC at high temperatures. According to the SEM measurement results, the lightweight aggregate had cracks inside of it at high temperatures, but the normal aggregate showed no damage. However, the ITZ degradation between the paste and the lightweight aggregate was improved compared to the normal aggregate. This result indicates that the degree of ITZ damage between pastes and aggregates significantly impacts UPV in concrete subjected to high temperatures compared with the effect of aggregates [14,44].

#### 3.1.4. Elastic Modulus on NC and LC after High Temperature

**Figure 11:**
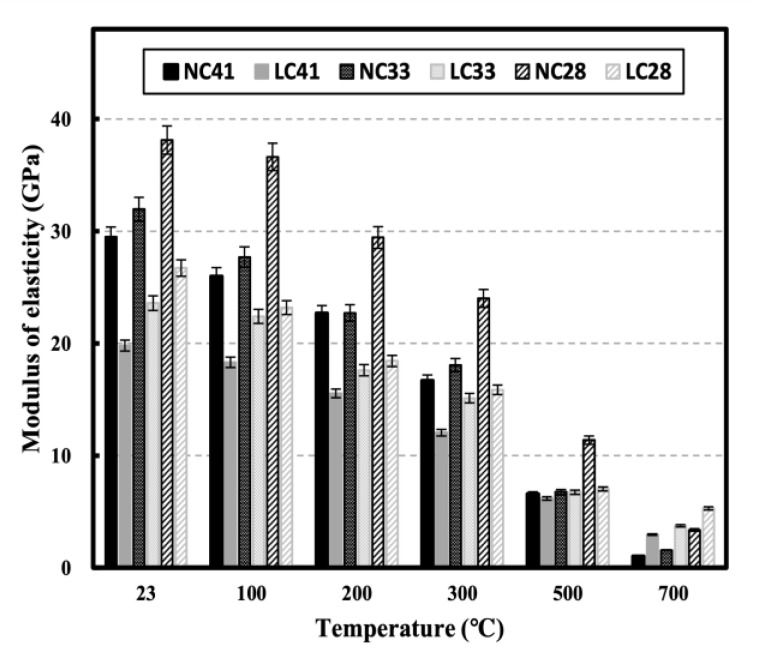
Elastic modulus of NC and LC after high temperature.

Figure 11 shows the elastic modulus of NC and LC in relation to temperature. At room temperature (20 °C), the elastic modulus between NC and LC at all W/C ratios significantly varied because the type and stiffness of concrete aggregates significantly affect the elastic modulus. The elastic modulus of NC was higher than that of LC at most temperatures, but at 700 °C, the elastic modulus of LC was higher.

**Figure 12:**
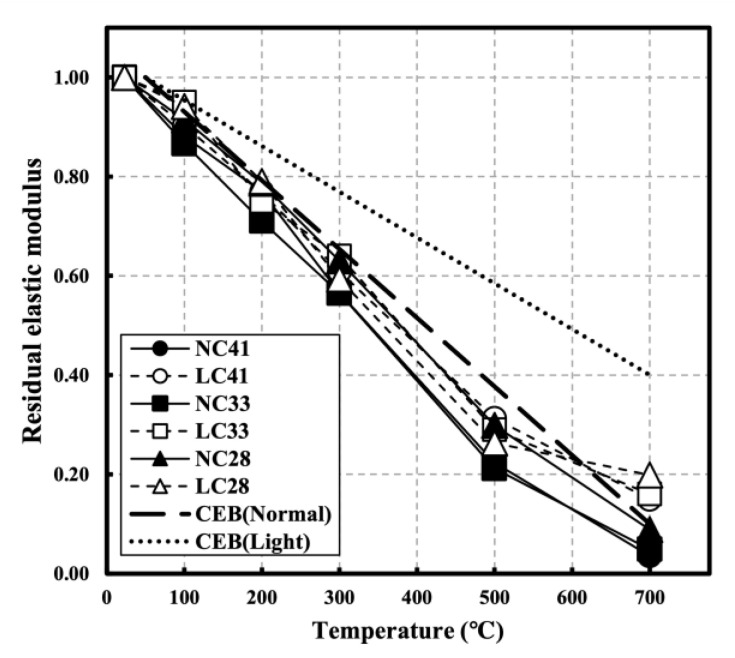
Residual elastic modulus of NC and LC after high temperature.

Figure 12 shows the residual elastic modulus of NC and LC in relation to temperature. Unlike compressive strength, the elastic modulus decreased linearly with temperature. Roufael et al. and Toric et al. also reported a linear decrease in the elastic modulus with increasing temperature [14,45]. The residual elastic modulus showed a lower residual rate than the residual compressive strength and residual UPV at the same temperature. At 500 °C, the residual compressive strength and residual UPV showed average values of 0.62 and 0.59, respectively, but the residual elastic modulus exhibited an average of 0.27. At 700 °C, the average residual compressive strength at all levels, except for NC41, was 0.42 and the average residual UPV was 0.49 for LC and 0.34 for NC; however, the average residual elastic modulus was 0.06 for NC and 0.17 for LC. According to Cakir et al., the reduction rate of the elastic modulus is higher than that of the compressive strength [38]. As the temperature increased, the residual elastic modulus of the LC was higher than that of the NC for all specimens, possibly caused by the material properties of the aggregate, particularly the compressive strength at high temperatures [37].

### 3.2. Correlation between Compressive Strength and Ultrasonic Pulse Velocity on NC and LC after High Temperature

Following exposure to high temperatures, a strong correlation was observed between the compressive strength of concrete and UPV in the form of a linear function. The influence of UPV, which linearly decreases along with an increase in temperature, is deduced as significant, and several strength prediction models in the form of a linear function have also been proposed based on the correlation between compressive strength and UPV at high temperatures [44,46,47,48].

**Figure 13:**
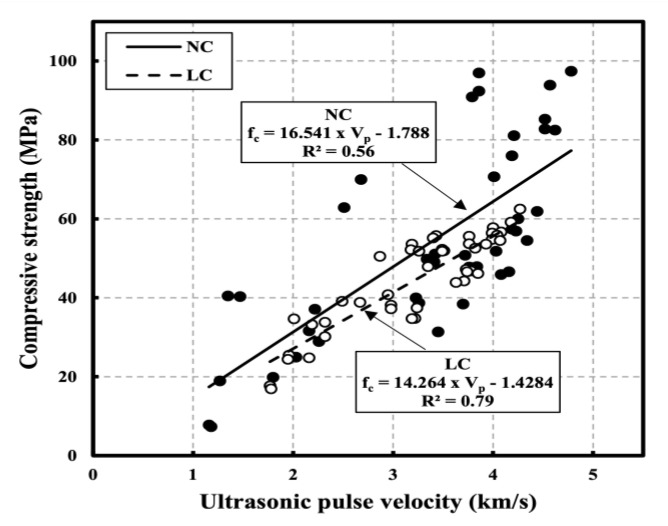
Correlation between compressive strength and UPV according to all W/C on NC and LC [22].

Figure 13 shows the correlation between compressive strength and UPV according to all W/C on NC and LC. Black dots mean NC and white dots mean LC. Most of the existing strength prediction equations proposed by researchers do not consider the W/C ratio. The analysis results without considering the W/C ratio are shown in Figure 13.

Insufficient accuracy is observed in relatively high strength and UPV ranges, and the correlation coefficient (R^2^), which is 0.56 for NC and 0.79 for LC, is also weak. Considering the quantities of the mixed materials are different depending on the design compressive strength, the characteristics of the mixture at high temperatures are also different. Therefore, it is deduced that different prediction equations must be used considering the W/C ratio for accurate strength prediction following exposure to high temperatures.

**Figure 14:**
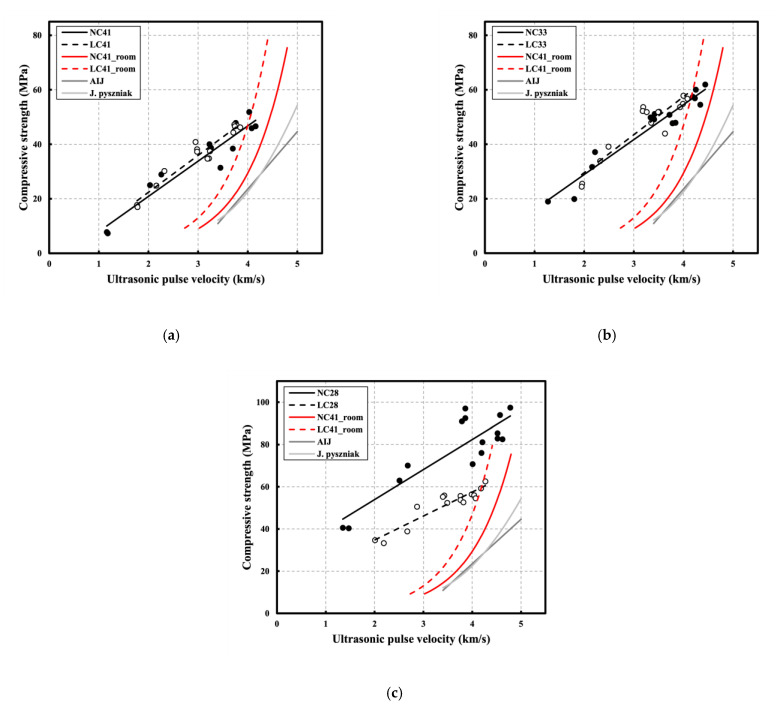
Correlation between compressive strength and UPV according to all each W/C on NC and LC: (**a**) W/C 41; (**b**) W/C 33; (**c**) W/C 28.

Figure 14 shows the correlation between compressive strength and UPV according to each W/C on NC and LC, while Table 6 summarizes the prediction equations and R^2^. Furthermore, the existing prediction equations were compared with the strength prediction equations using UPV analysis on the same specimen at room temperature (20 °C) [16]. Overall, high R^2^ values were observed, but relatively low R^2^ was observed at a W/C ratio of 0.28. It was determined that the irregular internal cracks and pores caused by the chemical decay of cement components following exposure to high temperatures significantly affected NC28 and LC28 with relatively high cement contents. Additionally, it appears that NC28 had the lowest R^2^ value because it was somewhat difficult to measure and analyze the significant strength change owing to ITZ degradation, micro-cracks from serious matrix collapse, and the influence of aggregates through UPV.

As the W/C ratio decreased, the NC and LC graphs increased, caused by the influence of high compressive strength and UPV as the W/C ratio decreased. At W/C ratios of 0.41 and 0.33, LC’s graph exceeded that of NC, possibly because of the aggregate used in LC’s high residual compressive strength and residual UPV (Figure 7 and Figure 9). However, NC28’s graph exceeded that of LC28 with a significant difference, possibly because the aggregate used in the LC developed low strength and low residual strength owing to its limited stiffness despite the same W/C ratio. Thus, it appears that R^2^ of NC was lower than that of LC in Figure 13, owing to the relatively large difference in the strength of NC depending on the W/C difference. The existing strength prediction equations showed significantly different results from those of the strength prediction equations using UPV analysis for the same specimen at room temperature (20 °C). The existing prediction equations at room temperature (20 °C) analyzed the mechanism of concrete strength development through measurement with UPV. However, the equations for predicting the strength of concrete following exposure to high temperatures are determined to be somewhat different because they analyze the degradation process mechanisms, such as cracks and pores, inside the concrete caused by high-temperature and cooling processes through measurement with UPV.

**Figure 15:**
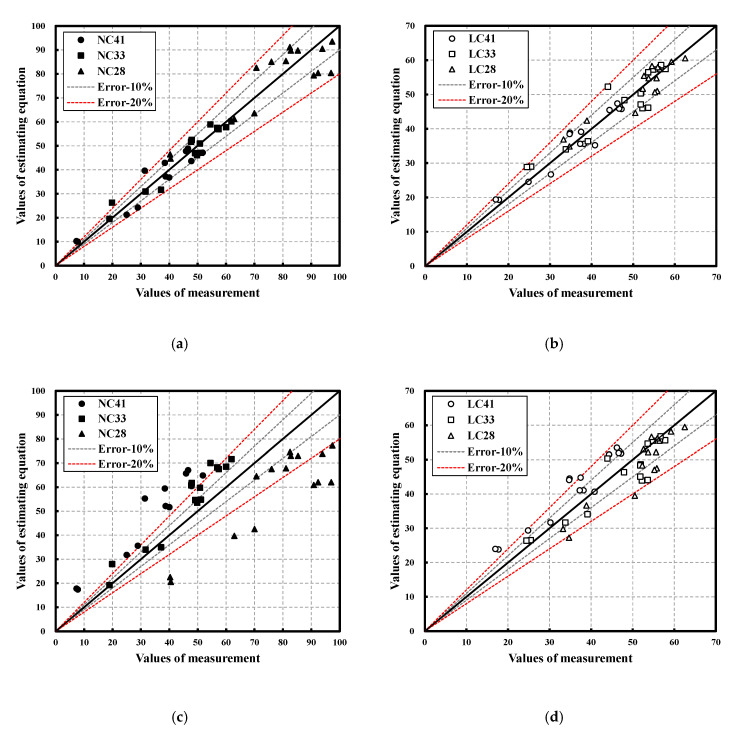
Error range between values of measurement and estimating equation: (**a**) each W/C of NC; (**b**) each W/C of LC; (**c**) all W/C of NC; (**d**) all W/C of LC.

Figure 15 shows the error range between values of measurement and estimating equation. Figure 15a,b show the error ranges of NC and LC, considering the W/C ratio, whereas Figure 15c,d show the error ranges of NC and LC without considering the W/C ratio. When the W/C ratio was not considered, data were severely scattered. Therefore, for accurate prediction of the residual strength of concrete through UPV following exposure to high temperatures, it is recommended to use the proposed prediction equations considering the aggregate type and W/C ratio.

## 4. Conclusions

This study examined the physical and mechanical properties of NC and LC at various strengths following exposure to high temperatures. The experimental results are summarized as follows.

1. After exposure to high temperatures, the highest mass loss increase rate was observed in the 100–300 °C temperature range. At 300 °C, the mass loss was approximately 5% for NC and 10% for LC. At 700 °C, the mass loss was approximately 9.29% for NC and 13.76% for LC, possibly caused by the influence of the water content inside the aggregate.
2. All specimens showed slight strength recovery after strength degradation in the temperature range below 300 °C and showed consistent strength degradation above 300 °C. At 700 °C, the residual compressive strengths of NC41, NC33, and NC28 were 0.15, 0.32, and 0.45, respectively, while those of LC41, LC33, and LC28 were 0.36, 0.45, and 0.53, respectively, possibly caused by the difference in the thermal expansion between the coarse aggregates.
3. All specimens showed continuous ultrasonic pulse velocity (UPV) as the temperature increased. At 700 °C, the residual UPVs of NC41, NC33, and NC28 were 0.29, 0.41, and 0.32, respectively, while those of LC41, LC33, and LC28 were 0.62, 0.61, and 0.67, respectively.
4. As the temperature increased, the elastic modulus continuously decreased at all levels and demonstrated a low residual rate compared with other mechanical properties. At 500 °C, the residual elastic modulus averaged 0.27 for all levels. At 700 °C, the average residual elastic moduli were approximately 0.06 for NC and 0.17 for LC.
5. When the correlation between the compressive strength of concrete and UPV following exposure to high temperatures was analyzed, the prediction equations that consider the water-binder (W/C) ratio had high R^2^; however, relatively low R^2^ was observed at a W/C ratio of 0.28. Additionally, the existing prediction equations at room temperature (20 °C) and the prediction equations using UPV analysis showed significant differences. Therefore, prediction equations that reflect high-temperature characteristics must be used for accurately predicting of the strength of concrete through UPV following exposure to high temperatures. Furthermore, it is necessary to consider the aggregate and W/C ratio of concrete.

In subsequent studies, it is necessary to conduct a study to accurately evaluate the residual strength of concrete subjected to high temperature in various strength ranges by adding experiments of several W/B ratios. In addition, it is judged that it is necessary to study the prediction of concrete strength using UPV according to various types of aggregates.

## Figures and Tables

**Table 1:** Physical properties of the materials [16].

Materials	Properties
Cement	Type|ordinary Portland cement Density: 3150 kg/m^3^, Fineness: 320 m^2^/kg
Coarse aggregate	Crushed granite aggregate Density: 2680 kg/m^3^, Fineness modulus: 7.03 Absorption: 0.68%, Maximum size: 20 mm
	Coal ash aggregate Density: 1470 kg/m^3^, Fineness modulus: 6.39 Absorption: 8.68%, Maximum size: 20 mm
Fine aggregate	River sand Density: 2540 kg/m^3^, Fineness modulus: 2.54 Absorption: 1.6%
Admixture	Polycarboxylic-based acid

**Table 2:** Chemical properties of cement [17].

Materials	Chemical Composition (%)								L.O.I ^2^
	CaO	SiO_2_	Al_2_O_3_	Fe_2_O_3_	MgO	SO_3_	K_2_O	Others	
OPC ^1^	60.34	19.82	4.85	3.30	3.83	2.88	1.08	0.86	3.02

^1^ OPC: Ordinary Portland cement. ^2^ L.O.I: Loss on ignition.

**Table 3:** Experimental program.

Classification	Estimation Equation
Specimen dimension	Φ100 × 200 mm
Type of coarse aggregate	Crushed granite aggregate, Coal ash aggregate
Design compressive strength	30, 45, 60 MPa
Curing conditions	Water, Room temperature: 20 ± 2 °C, Humidity: 60 ± 5%
Temperature	23, 100, 200, 300, 500, 700 °C
Heating rate	1 °C/min
Temperature	60 min
Test items	Mass loss (%), Compressive strength (MPa), Ultrasonic pulse velocity (km/s), Elastic modulus (GPa)

**Table 4:** Mix proportions of NC and LC.

MIX ID	f_ck_ (MPa)	W/C ^1^	S/a ^2^ (%)	Unit Weight (kg/m^3^)			
				W ^3^	C ^4^	S ^5^	G ^6^
LC41	30	0.41	46.0	165	400	799	758
NC41						799	956
LC33	45	0.33	43.0		500	711	762
NC33						711	961
LC28	60	0.28	43.0		600	676	724
NC28						676	913

^1^ W/C: water/binder. ^2^ S/a: sand/aggregate. ^3^ W: water. ^4^ C: cement. ^5^ S: sand. ^6^ G: gravel.

**Table 5:** Testing of mechanical properties [22].

Test Items	Test Method	
Compressive strength (MPa)	ASTM C39/C39M	(1) $$V_{p}=\frac{L}{t}$$ $V_{p}$: ultrasonic pulse velocity(m/s) L: distance(m) t: time(s)
Elastic modulus (GPa)	ASTM C469	
Ultrasonic Pulse Velocity (km/s)	ASTM C597	

**Table 6:** Correlation between compressive strength and UPV.

ID	Equation	Correlation Coefficient (R^2^)
NC41	$f_{c}=12.941\;\times$ V_p_ − 5.0134	R^2^ = 0.92
LC41	$f_{c}=13.508\;\times$ V_p_ − 4.6244	R^2^ = 0.93
NC33	$f_{c}=12.83\;\times$ V_p_ + 3.1922	R^2^ = 0.93
LC33	$f_{c}=13.944\;\times$ V_p_ + 1.6587	R^2^ = 0.85
NC28	$f_{c}=14.224\;\times$ V_p_ + 25.482	R^2^ = 0.77
LC28	$f_{c}=11.38\;\times$ V_p_ + 11.986	R^2^ = 0.87

## Data Availability

Data presented in this study are available on request from the corresponding author.
